# Editorial: Carbon allocation, volume II

**DOI:** 10.3389/fpls.2023.1342494

**Published:** 2023-11-29

**Authors:** Rezwan Tanvir, Susan I. Gibson, Eve Syrkin Wurtele, Ling Li

**Affiliations:** ^1^ Department of Biological Sciences, Mississippi State University, Mississippi State, MS, United States; ^2^ Department of Plant & Microbial Biology, University of Minnesota Twin Cities, Saint Paul, MN, United States; ^3^ Department of Genetics, Development and Cell Biology, Iowa State University, Ames, IA, United States

**Keywords:** carbon flux, sugar accumulation/transport/metabolism, stress response, miRNA, single-cell studies, transcriptional regulation, crop improvement

## Introduction

The foundational elements of cellular architecture and metabolism in all living organisms are woven from carbon building blocks, underscoring the pivotal role of efficient carbon allocation in the survival and growth of all organisms. For photosynthetic entities, the intricacies of maintaining carbon flux introduce additional layers of complexity. To unravel the underlying mechanisms governing carbon storage and transfer in plants, a thorough comprehension of carbon allocation regulation becomes paramount. This is key in deciphering the multifaceted impact of carbon allocation on various aspects of plant biology, such as growth, development, reproduction, defense mechanisms, yield, biomass production, and numerous other traits (Qi et al., 2019; Hartmann et al., 2020; O’Conner et al., 2021; Boatwright et al., 2022; Tanvir et al., 2022a; Tanvir et al., 2022b; Wang et al., 2023). Despite significant strides in our comprehension of plant carbon metabolism in recent decades, the regulatory mechanisms governing carbon allocation remain elusive, primarily due to the sophisticated nature of key components involved in carbon fluxes.

Plants harness the power of sunlight to drive photosynthesis. The resulting carbon is then distributed among various plant organs to fulfill both structural and metabolic needs. Primarily stored as starch and soluble sugars, this allocation ensures sustenance during nighttime and periods of carbon scarcity (MacNeill et al., 2017; Schiestl-Aalto et al., 2019; Kannenberg and Phillips, 2020; Tsamir‐Rimon et al., 2021). Diverse mechanisms regulate carbon allocation, encompassing transcriptional regulation of genes associated with carbon uptake, transportation and storage, as well as factors such as nutrient availability, homeostasis, redox status, environmental perturbation, and stress (Chaput et al., 2020; Huang et al., 2021; Keller et al., 2021; Ouyang et al., 2021; Wei et al., 2022). The convoluted interplay of these mechanisms with other signaling and metabolic networks constitutes a captivating yet challenging area of study, essential for enhancing our scientific understanding and evaluating potential biotechnological implications.

Within this Research Topic, we underscore the importance of fostering multidisciplinary investigations that integrate diverse approaches across multiple pathways in carbon uptake, transport, conversion, and utilization. Spanning from single-cell studies to on-field research, these investigations delve into the molecular complexity of these mechanisms, seeking to enhance our understanding and explore practical applications. The Research Topic comprises five original research papers that investigated transcriptional regulation to modify soybean seed composition, enhance sugar accumulation and transport in the leaf and stem of sorghum, utilize single-cell models to explore an orphan gene network related to carbon-nitrogen allocation and identify miRNAs involved in sucrose stress response in Arabidopsis.

## What have we learned from this Research Topic?

Carbon allocation plays a pivotal role in shaping the composition of soybean seeds, with an intricate balance that often involves trade-offs in carbon distribution. The study by Aznar-Moreno et al. on *SUGAR-DEPENDENT1* (*SDP1*) provides a model for how suppression of *SDP1* can redistribute carbon from specific undigestible carbohydrates to triacylglycerol without adversely affecting protein content, presenting a promising avenue to enhance the nutritional quality of soybeans.

In a separate study, McKinley et al. characterized the expression profiles of genes associated with the *myo*-inositol and raffinose family oligosaccharides (RFOs) pathways in bioenergy sorghum, particularly focusing on sugar transport and accumulation. The study revealed alpha-galactosidases (AGAs) exhibited significant induction during the stem sucrose accumulation phase, suggesting a potential role in accumulating non-structural carbohydrates (NSCs) in sorghum stems. The research provides valuable insights into the molecular complexity of the *myo*-inositol-RFO pathway in bioenergy sorghum, highlighting its potential contributions to stress tolerance, sugar transport, and stem sucrose accumulation.

The orphan gene *QQS*, a unique carbon flux regulator exclusive to Arabidopsis, exerts influence over carbon and nitrogen allocation when introduced into other plant species. QQS interacts with the NF-YC subunit of Nuclear Factor Y (Li et al., 2015). Wang et al. utilizing genetically simpler organisms—yeast (*Saccharomyces cerevisiae*) and the green alga (*Chlamydomonas reinhardtii*)— systematically dissected the effects of *QQS* on carbon and nitrogen allocation. Their findings suggest that QQS can alter carbon and nitrogen allocation by interacting with NF-YC in *C. reinhardtii* (lacking an NF-YA subunit), and in *S. cerevisiae*. This research highlights how orphan genes like *QQS* can provide alternative, rapid evolutionary forces contributing to the emergence of novel phenotypes alongside the slower, adaptive evolution described by Darwinian principles (Arendsee et al., 2014; Singh and Wurtele, 2020). It provides a model for investigating the functional mechanisms of novel carbon flux regulators.

A global transcriptomic analysis focusing on genes involved in sugar transport, sucrose metabolism, and other regulatory processes governing the spatiotemporal accumulation of sugars in sorghum stems post-decapitation was conducted by Xue et al. The objective was to understand how alterations in the source-sink relationships impact regulators and transporters to enhance sugar accumulation. Downregulation of *SbbHLH093* resulted in delayed reproductive development and an extended period of sugar accumulation. Sorghum decapitation also led to the downregulation of the *Dry* (*D*) locus gene associated with programmed cell death (PCD), ensuring prolonged stem development. Additionally, upregulation of sugar transporter genes such as *SbSWEET1A* in phloem companion cells positively contributed to sugar accumulation. These findings offer opportunities to boost sugar yield in bioenergy crops.

In a study by Azad et al., the involvement of microRNAs (miRNAs) in response to excess sucrose was investigated, with a specific focus on potential connections between miRNAs and reactive oxygen species (ROS) production and anthocyanin biosynthesis pathways, employing deep sequencing in Arabidopsis. The research revealed that ROS signaling is intricately linked to anthocyanin production through *Pentatrico Peptide Repeat* (*PPR*) genes. Notably, two novel non-canonical targets affected by excess sucrose were identified: miR408 (targeting *Flavonoid 3’-Hydroxylase*) and miR398b* (targeting *ORANGE*). This investigation provides valuable insights into the complex interplay among sucrose signaling, miRNA regulation, and various plant metabolic pathways.

In summary, the articles featured in this Research Topic collectively underscore the significance of carbon flux in plant development, growth, and the maintenance and regulation of primary and specialized metabolic pathways. The Research Topic emphasizes the critical importance of understanding carbon allocation, not only for advancing our scientific knowledge but also for driving future progress in sustainable agriculture, crop improvement and productivity, and biomass and bioenergy production.

## Author contributions

RT: Writing – original draft, Writing – review & editing. SG: Writing – review & editing. EW: Writing – review & editing. LL: Writing – review & editing, Writing – original draft.

## References

[B1] Arendsee Z. W. Li L. Wurtele E. S. (2014). Coming of age: orphan genes in plants. Trends Plant Sci. 19 (11), 698–708. doi: 10.1016/j.tplants.2014.07.003 25151064

[B2] Boatwright J. L. Sapkota S. Myers M. Kumar N. Cox A. Jordan K. E.. (2022). Dissecting the genetic architecture of carbon partitioning in sorghum using multiscale phenotypes. Front. Plant Sci. 13. doi: 10.3389/fpls.2022.790005 PMC9159972 35665170

[B3] Chaput V. Martin A. Lejay L. (2020). Redox metabolism: the hidden player in carbon and nitrogen signaling? J. Exp. Bot. 71 (13), 3816–3826. doi: 10.1093/jxb/eraa078 32064525

[B4] Hartmann H. Bahn M. Carbone M. Richardson A. D. (2020). Plant carbon allocation in a changing world–challenges and progress: introduction to a Virtual Issue on carbon allocation. New Phytol. 227 (4), 981–988. doi: 10.1111/nph.16757 32662104

[B5] Huang J. Hammerbacher A. Gershenzon J. Van Dam N. M. Sala A. McDowell N. G.. (2021). Storage of carbon reserves in spruce trees is prioritized over growth in the face of carbon limitation. Proc. Natl. Acad. Sci. 118 (33), e2023297118. doi: 10.1073/pnas.2023297118 34389667 PMC8379986

[B6] Kannenberg S. A. Phillips R. P. (2020). Non-structural carbohydrate pools not linked to hydraulic strategies or carbon supply in tree saplings during severe drought and subsequent recovery. Tree Physiol. 40 (2), 259–271. doi: 10.1093/treephys/tpz132 31860721

[B7] Keller I. Rodrigues C. M. Neuhaus H. E. Pommerrenig B. (2021). Improved resource allocation and stabilization of yield under abiotic stress. J. Plant Physiol. 257, 153336. doi: 10.1016/j.jplph.2020.153336 33360492

[B8] Li L. Zheng W. Zhu Y. Ye H. Tang B. Arendsee Z. W.. (2015). *QQS* orphan gene regulates carbon and nitrogen partitioning across species via NF-YC interactions. Proc. Natl. Acad. Sci. 112 (47), 14734–14739. doi: 10.1073/pnas.1514670112 26554020 PMC4664325

[B9] MacNeill G. J. Mehrpouyan S. Minow M. A. Patterson J. A. Tetlow I. J. Emes M. J. (2017). Starch as a source, starch as a sink: the bifunctional role of starch in carbon allocation. J. Exp. Bot. 68 (16), 4433–4453. doi: 10.1093/jxb/erx291 28981786

[B10] O’Conner S. Zheng W. Qi M. Kandel Y. Fuller R. Whitham S. A.. (2021). GmNF-YC4-2 increases protein, exhibits broad disease resistance and expedites maturity in soybean. Int. J. Mol. Sci. 22 (7), 3586. doi: 10.3390/ijms22073586 33808355 PMC8036377

[B11] Ouyang S.-N. Gessler A. Saurer M. Hagedorn F. Gao D.-C. Wang X.-Y.. (2021). Root carbon and nutrient homeostasis determines downy oak sapling survival and recovery from drought. Tree Physiol. 41 (8), 1400–1412. doi: 10.1093/treephys/tpab019 33595075 PMC8436808

[B12] Qi M. Zheng W. Zhao X. Hohenstein J. D. Kandel Y. O’Conner S.. (2019). *QQS* orphan gene and its interactor *NF-YC4* reduce susceptibility to pathogens and pests. Plant Biotechnol. J. 17 (1), 252–263. doi: 10.1111/pbi.12961 29878511 PMC6330549

[B13] Schiestl-Aalto P. Ryhti K. Mäkelä A. Peltoniemi M. Bäck J. Kulmala L. (2019). Analysis of the NSC storage dynamics in tree organs reveals the allocation to belowground symbionts in the framework of whole tree carbon balance. Front. For. Glob. Change 2. doi: 10.3389/ffgc.2019.00017

[B14] Singh U. Wurtele E. S. (2020). How new genes are born. Elife 9, e55136. doi: 10.7554/eLife.55136 32072921 PMC7030788

[B15] Tanvir R. Ping W. Sun J. Cain M. Li X. Li L. (2022a). *AtQQS* orphan gene and *NtNF-YC4* boost protein accumulation and pest resistance in tobacco (*Nicotiana tabacum*). Plant Sci. 317, 111198. doi: 10.1016/j.plantsci.2022.111198 35193747

[B16] Tanvir R. Wang L. Zhang A. Li L. (2022b). Orphan genes in crop improvement: Enhancing potato tuber protein without impacting yield. Plants (Basel) 11 (22), 3076. doi: 10.3390/plants11223076 36432805 PMC9696052

[B17] Tsamir-Rimon M. Ben-Dor S. Feldmesser E. Oppenhimer-Shaanan Y. David-Schwartz R. Samach A.. (2021). Rapid starch degradation in the wood of olive trees under heat and drought is permitted by three stress-specific beta amylases. New Phytol. 229 (3), 1398–1414. doi: 10.1111/nph.16907 32880972

[B18] Wang K. Yan J. Tanvir R. Li L. Liu Y. Zhang W. (2023). Improved forage quality and biomass yield of alfalfa (*Medicago sativa* L.) by Arabidopsis *QQS* orphan gene. Curr. Plant Biol. 35, 100295. doi: 10.1016/j.cpb.2023.100295

[B19] Wei S. Li X. Lu Z. Zhang H. Ye X. Zhou Y.. (2022). A transcriptional regulator that boosts grain yields and shortens the growth duration of rice. Science 377 (6604), eabi8455. doi: 10.1126/science.abi845 35862527

